# Identification and Antibiotic Profiling of *Wohlfahrtiimonas chitiniclastica*, an Underestimated Human Pathogen

**DOI:** 10.3389/fmicb.2021.712775

**Published:** 2021-09-22

**Authors:** Anna Kopf, Boyke Bunk, Sina M. Coldewey, Florian Gunzer, Thomas Riedel, Percy Schröttner

**Affiliations:** ^1^Institute of Medical Microbiology and Virology, University Hospital Carl Gustav Carus, Dresden, Germany; ^2^Leibniz Institute DSMZ-German Collection of Microorganisms and Cell Cultures GmbH, Braunschweig, Germany; ^3^Clinic for Anaesthesiology and Intensive Care Medicine, Jena University Hospital, Jena, Germany; ^4^Septomics Research Center, Jena University Hospital, Jena, Germany; ^5^Department of Hospital Infection Control, University Hospital Carl Gustav Carus, Dresden, Germany; ^6^German Center for Infection Research (DZIF), Partner Site Hannover-Braunschweig, Braunschweig, Germany

**Keywords:** *Wohlfahrtiimonas chitiniclastica*, antibiotic profiling, MALDI-TOF MS, 16S rRNA, primer, VITEK 2, digital DNA-DNA hybridization

## Abstract

In the past 12 years, several case reports have clearly demonstrated that *Wohlfahrtiimonas chitiniclastica* is capable of causing sepsis and bacteremia in humans. However, since most clinicians are not familiar with this species, little is known about its pathogenicity and treatment options while it is as rare but underestimated human pathogen. Therefore, a larger strain collection is required so that methods can be identified that are most suitable to obtain rapid and reliable identification. Moreover, the antimicrobial resistance profile needs to be elucidated in order to explore possible treatment options. Over a period of 6 years, we therefore have collected a total of 14 *W. chitiniclastica* isolates in routine diagnostics, which now served as the basis for a comprehensive characterization with respect to identification and antibiotic profiling. We compared the accuracy and convenience of several identification techniques in which MALDI-TOF MS and sequencing of the 16S rRNA gene have proven to be suitable for identification of *W. chitiniclastica.* In addition, whole genome sequencing (WGS)-based digital DNA-DNA hybridization (dDDH) was used as a reference method for strain identification, and surprised with the detection of a novel *W. chitiniclastica* subspecies. A combination of *in silico* and *in vitro* analyses revealed a first insight into the antimicrobial resistance profile and the molecular basis of antimicrobial resistance. Based on our findings, trimethoprim/sulfamethoxazole, levofloxacin, and cephalosporins (e.g., ceftazidime) may be the best antibiotics to use in order to treat infections caused by *W. chitiniclastica*, while resistance to fosfomycin, amikacin and tobramycin is observed.

## Introduction

The gammaproteobacterium *Wohlfahrtiimonas chitiniclastica* has first been isolated from larvae of *Wohlfahrtia magnifica* (Tóth et al., 2008), an obligatory parasitic fly that causes myiasis by depositing eggs and larvae in mammalian wounds both in animals and humans (Robbins and Khachemoune, 2010). Bacteria belonging to this species are described as Gram-negative, strictly aerobic, and non-motile rods. Furthermore, the organism is catalase and oxidase positive, while biochemical tests for urease, indole, and H_2_S are negative (Tóth et al., 2008; Schröttner et al., 2017). They are also noted to have strong chitinase activity, which may be an indicator for a symbiotic relationship with its host fly while playing an important role in metamorphosis (Schröttner et al., 2017; Snyder et al., 2020).

In April 2021, GenBank (Benson et al., 2013) lists 12 genomes of *W. chitiniclastica* strains while three draft genome reports were published (Cao et al., 2013; Zhou et al., 2016; Matos et al., 2019). The strains are described to be susceptible to the majority of known antibiotics with the exception of fosfomycin (Schröttner et al., 2017; Matos et al., 2019). First genome annotations revealed genes coding for macrolide-specific efflux pumps (*macA* and *macB*) (Matos et al., 2019) and a *bla*_*VEB*__–__1_ gene cassette which confers resistance to ceftazidime, ampicillin, and tetracycline (Zhou et al., 2016). However, in-depth analysis is still required to generate a comprehensive antimicrobial resistance profile as the available antimicrobial susceptibility data are mostly based on case reports and preliminary genome annotations (Zhou et al., 2016; Schröttner et al., 2017; Matos et al., 2019).

**FIGURE 1:**
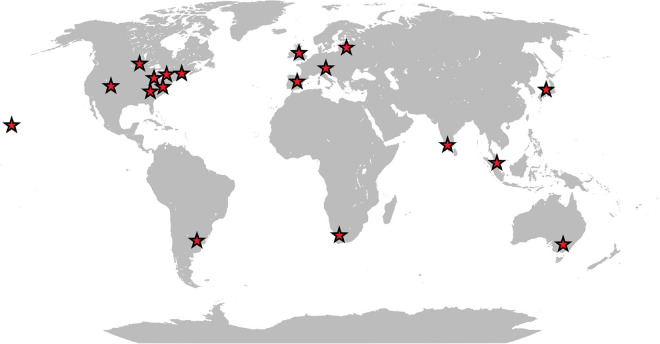
Schematic map showing the geographic distribution of reported human infections caused by *W. chitiniclastica*.

Originally isolated from a homogenate of fly larvae, there is increasing evidence that *W. chitiniclastica* may be the cause of several diseases in humans. Although the pathogenesis of *W. chitiniclastica* is not yet fully understood, the bacterium is expected to enter traumatic skin lesions through fly larvae, resulting in severe myiasis and/or wound contamination (Robbins and Khachemoune, 2010; Thaiwong et al., 2014; Schröttner et al., 2017). To date, 23 human case reports from 18 countries across the globe have been published (Table 1 and Figure 1) indicating *W. chitiniclastica* to be associated with humans sepsis and bacteremia. For example, Almuzara et al. (2011) reported the first case of fulminant sepsis with fatal outcome and Campisi et al. (2015) observed *W. chitiniclastica* bacteremia associated with myiasis, to name but a few. However, since most clinicians are not familiar with this species, it can be assumed that *W. chitiniclastica* has hardly been recognized as a possible cause while it has recently been described as a new underestimated human pathogen (Schröttner et al., 2017). Therefore, it is necessary to initiate systematic investigations to gain more knowledge about its virulence and treatment options. For this reason, first methods need to be identified that are most suitable to obtain a fast, reliable and robust species identification. Moreover, we need to shed light on the antimicrobial resistance profile to gain knowledge about primary resistances in order to treat infections successfully. In this study, we therefore compared the accuracy of several routine methods of bacterial identification and performed antimicrobial susceptibility testing of 14 isolates collected from clinical samples. Additionally, we conducted whole genome data to elucidate the molecular basis of antimicrobial resistance and to confirm correct species designation.

**TABLE 1:** Current overview of cases of human infection and colonization with *W. chitiniclastica.*

**Case**	**Year**	**Age**	**Gender**	**Region**	**Underlying disease(s)/reason for hospital admission**	**Social conditions**	**Insect larvae/infected wounds**	**Antibiotic treatment**	**Outcome**	**References**
1	2009	60	f	Marseille, France	Fatigue	Homeless, poor hygienic conditions, alcoholism	Positive	Ceftriaxone	Survived	Rebaudet et al., 2009
2	2011	70	m	Buenos Aires, Argentina	Occlusive peripheral arteriopathy of the lower limbs/sensory impairment	Homeless, history of alcoholism and smoking	Negative	Ciprofloxacin, Ampicillin, Ceftazidime, Amikacin	Fatal	Almuzara et al., 2011
3	2015	82	f	Guildford, United Kingdom	Recurrent falls, hypertension, chronic kidney disease, ischemic heart disease, hypercholesterolemia, osteoarthritis/found unconscious	NP	Positive	Cefuroxime, Clarithromycin, Flucloxacillin	Survived	Campisi et al., 2015
4	2015	26	m	Salt Lake City, United States	Morbid obesity, lymphoedema, cellulitis/progressive gangrenous changes	NP	NP	Cefpodoxime	Survived	de Dios et al., 2015
5	2015	64	m	Tartu, Estonia	Gangrene in distal parts of the legs and amputation of the feet/admission due to an accident	Alcoholism	NP	Amoxicillin/Clavulanate	Survived	Kõljalg et al., 2015
6	2015	43	m	Trivandrum, India	Diabetes, deep ulcer, cellulitis, gangrene/progressing gangrenous changes	Alcoholism, smoking	NP	Cefoperazone/Sulbactam, Cefpodoxime	Survived	Suryalatha et al., 2015
7	2016	17	m	Cape Town, South Africa	Soft-tissue infection due to an accident	Good hygienic conditions	Negative	Ceftriaxone	Survived	Hoffmann et al., 2016
8	2016	72	m	Hawaii, United States	Stroke, found unconscious	Poor hygienic conditions	Positive	Piperacillin/Tazobactam, Clindamycin, Vancomycin	Fatal	Nogi et al., 2016
9	2016	69	f	Hawaii, United States	Ruptured cerebral aneurysm and right hemiparesis/sacral pain and painful urination	Homeless, poor hygienic conditions	Negative	Ceftaroline fosamil, Meropenem	Survived	Nogi et al., 2016
10	2017	41	f	Ohio, United States	Abdominal pain, stage IV right ischial decubitus ulcer, bilateral leg lymphedema, congenital lumbar myelomeningocele causing paraplegia post spinal fixation	Poor hygienic conditions	Negative	Vancomycin, Cefepime, Metronidazol	Fatal	Chavez et al., 2017
11	2017	47	f	Malaysia	Metastatic colorectal adenocarcinoma, immunosuppression	Good personal hygiene	Negative	Cefoperazone	Fatal	Suraiya et al., 2017
12	2017	79	m	Dresden, Germany	Diabetes mellitus, coronary heart disease, chronic renal failure, venous insufficiency/progressive ulceral disease	Normal social conditions	Negative	Cefuroxime, Levofloxacin	Survived	Schröttner et al., 2017
13	2017	43	m	Dresden, Germany	Alcoholism/treatment of alcohol withdrawal syndrome	Homeless, alcoholism, ulceral disease	Negative	No antibiotic treatment	Survived	Schröttner et al., 2017
14	2017	78	f	Dresden, Germany	Severe obesity, chronic venous insufficiency, arterial hypertension, chronic heart failure NYHA II/progressive ulceral disease	Difficult social conditions	Negative	No antibiotic treatment	Survived	Schröttner et al., 2017
15	2017	71	m	Dresden, Germany	Deep vein thrombosis, leg ulcers/speech disorder as consequence of a tablet and alcohol intoxication	NP	Negative	No antibiotic treatment	Survived	Schröttner et al., 2017
16	2018	75	m	Tokyo, Japan	Squamous cell carcinoma, chronic wounds with maggots	NP	Positive	Cefepime and Metronidazole i.v.	Survived	Katanami et al., 2018
17	2018	57	m	Washington, United States	Right ankle wet gangrene, chronic cirrhosis, lung atelectasis	NP	Positive	NP	NP	Bonwitt et al., 2018
18	2018	37	m	Indiana, United States	Chronic lymphedema and ulcers of the lower left extremity presented with myiasis of the left foot and leg, myiasis	NP	Positive	Vancomycin, Clindamycin, Piperacillin/Tazobactam	Survived	Lysaght et al., 2018
19	2019	63	m	Kentucky, United States	Cardiac arrest, anoxic brain injury, foot ulcer containing maggots, cirrhosis	Poor hygienic conditions, alcoholism, tobacco abuse	Positive	Vancomycin i.v., Piperacillin/Tazobactam i.v.	Fatal	Fenwick et al., 2019
20	2019	87	f	Kentucky, United States	NP	Homeless, poor hygienic conditions,	NP	NP	NP	Fenwick et al., 2019
21	2019	54	m	Melbourne, Australia	Unconscious collapse at home, chronic inflammatory demyelinating polyneuropathy with severe sensory and motor neuropathy, alcohol dependence, and hereditary hemochromatosis	NP	Positive	Piperacillin/Tazobactam, Meropenem	Survived	Connelly et al., 2019
22	2020	82	m	Harrisburg, United States	Fall at home with associated confusion, mitral valve replacement due to mitral stenosis, peripheral vascular diseases	Poor hygienic conditions	Positive	Vancomycin, Cefepime, Daptomycin	Survived	Snyder et al., 2020
23	2021	70	m	Fargo, United States	B cell non-Hodgkin lymphoma, chronic left temporal wound	NP	Positive	Levofloxacin	Survived	Bueide et al., 2021

*f, female; m, male, NP, not provided.*

## Materials and Methods

### Collection of *W. chitiniclastica* Strains

Over a period of 6 years, a total of 14 *W. chitiniclastica* strains have been collected in routine diagnostics (see Table 2). All isolates were recovered exclusively from diagnostic cultures analyzed at the Institute for Medical Microbiology and Virology, University Hospital Carl Gustav Carus (Dresden, Germany). Prior to this publication the isolates DSM 100374, DSM 100374, DSM 100676, and DSM 100917 were briefly described as part of a review article (Schröttner et al., 2017); however a thorough analysis has not been performed. Subsequently, all strains were collected and stored in Pro-Lab Diagnostics^TM^ Microbank^TM^ (Fisher Scientific, Schwerte, Germany). The bacteria were additionally deposited at the “Open Collection” of the Leibniz Institute DSMZ-German Collection of Microorganisms and Cell Cultures (Braunschweig, Germany). The type strain DSM 18708^T^ was purchased from the DSMZ and included as reference strain in this study.

**TABLE 2:** Strain sources and patients’ clinical data.

**DSM number**	**Species**	**Gender**	**Age**	**Microbial spectrum**	**Additional information**
100374	*W. chitiniclastica*	m	43	*Proteus mirabilis*	Homeless, alcoholism, diabetic foot, MRSA screening, exclusion of tuberculosis
100375	*W. chitiniclastica*	m	78	*Escherichia coli*	Normal social conditions, diabetes mellitus, coronary heart disease, chronic renal failure, venous insufficiency/progressive ulceral disease
100676	*W. chitiniclastica*	m	78	*Staphylococcus aureus, Proteus mirabilis, Serratia marcescens, Myroides odoratimimus*	Difficult social conditions, diabetic foot, ulcus cruris, severe obesity, chronic venous insufficiency, arterial hypertension, chronic heart failure NYHA II/progressive ulceral disease
100917	*W. chitiniclastica*	m	72	*Proteus mirabilis, Providencia stuartii, Pseudomonas aeruginosa*	Diabetic foot, adiposity, thrombosis, thrombophlebitis, anticoagulation, speech disorder as consequence of a tablet, and alcohol intoxication in suicidal intent
105708	*W. chitiniclastica*	m	90	*Morganella morganii, Bacteroides fragilis*	Tumorous skin formation (head, neck)
105712	*W. chitiniclastica*	f	82	*Providencia rettgeri*, hemolytic *Streptococcus* Group G, *Proteus vulgaris*	Renal failure, ulcus cruris
105838	*W. chitiniclastica*	m	60	Coagulase negative *Staphylococcus, Aeromonas veronii, Klebsiella oxytoca*	Diabetic foot, MRSA screening
105839	*W. chitiniclastica*	m	84	*Proteus mirabilis*, hemolytic *Streptococcus* Group C	Diabetic foot, ulcus cruris
105984	*W. chitiniclastica*	m	60	Coagulase negative *Staphylococcus*, *Proteus hauseri, Klebsiella oxytoca*, rod-shaped *Corynebacterium spp.*	Diabetic foot
106597	*W. chitiniclastica*	m	60	Coagulase negative *Staphylococcus*, *Viridans-Streptococcus, Vagococcus fluvialis, Morganella morganii, Klebsiella oxytoca*	Diabetic foot
108048	*W. chitiniclastica*	m	75	*Staphylococcus aureus, Proteus penneri/vulgaris, Providencia rettgeri*	Type 2 diabetic
108045	*W. chitiniclastica*	m	65	*Staphlococcus aureus, Proteus vulgaris*	Diabetic foot
110179	*W. chitiniclastica*	m	60	*Staphylococcus sciuri, coagulase negative Staphylococcus, Viridans Streptococcus, Klebsiella oxytoca, Vagococcus fluvialis*	Diabetic foot
110473	*W. chitiniclastica*	m	43	*Proteus mirabilis, Klebsiella oxytoca, Providencia rettgeri, Staphylococcus aureus*	NP

*All isolates were recovered exclusively from diagnostic cultures analyzed at the Institute for Medical Microbiology and Virology, University Hospital Carl Gustav Carus (Dresden, Germany). All strains have been isolated from wound swab.*

*f, female; m, male; NP, not provided.*

### Identification of *W. chitiniclastica* Using VITEK 2

Frozen colonies were grown on Colombia blood agar plates (bioMérieux, Nürtingen, Germany) for 24 h at 37°C. A single colony from each isolate was picked and transferred to a new Colombia blood agar plate. After another incubation period of 24 h at 37°C, the colonies were suspended in a solution of 3 ml of 0.45% saline. A turbidity of 0.5–0.63 McFarland standard using VITEK DensiCHEK Plus (bioMérieux, Nürtingen, Germany) was established. Bacteria were identified with a VITEK 2 system (bioMérieux, Nürtingen, Germany) using GN ID cards (for analysis of gram-negative bacteria) as described in a previous study (Schröttner et al., 2014). Results are displayed in Table 3 and Supplementary Table 5.

**TABLE 3:** Comparison of diagnostic methods applied for identification of *W. chitiniclastica.*

**DSM number**	**MALDI-TOF MS identification results**	**VITEK 2 identification results**	**16S rDNA TPU1_RTU4**	**16S rDNA 27F_1492R**	**dDDH results**
100374	*W. chitiniclastica* (2.25)	*A. lwoffii* (96%)	*W. chitiniclastica* (99.9%)	*W. chitiniclastica* (99.7%)	*W. chitiniclastica* (74.8%)
100375	*W. chitiniclastica* (2.45)	*A. lwoffii* (99%)	*W. chitiniclastica* (99.9%)	*W. chitiniclastica* (99.7%)	*W. chitiniclastica* (74.3%)
100676	*W. chitiniclastica* (2.18)	*A. lwoffii* (96%)	*W. chitiniclastica* (99.7%)	*W. chitiniclastica* (99.8%)	*W. chitiniclastica* (74.6%)
100917	*W. chitiniclastica* (2.23)	*A. lwoffii* (96%)	*W. chitiniclastica* (99.9%)	*W. chitiniclastica* (99.3%)	*W. chitiniclastica* (74.6%)
105708	*W. chitiniclastica* (2.33)	*A. lwoffii* (96%)	*W. chitiniclastica* (100%)	*W. chitiniclastica* (99.4%)	*W. chitiniclastica* (74.1%)
105712	*W. chitiniclastica* (2.05)	*A. lwoffii* (96%)	*W. chitiniclastica* (99.9%)	*W. chitiniclastica* (99.6%)	*W. chitiniclastica* (75.0%)
105838	*W. chitiniclastica* (2.35)	*A. lwoffii* (96%)	*W. chitiniclastica* (99.2%)	*W. chitiniclastica* (99.7%)	*W. chitiniclastica* (75.1%)
105839	*W. chitiniclastica* (2.15)	*A. lwoffii* (96%)	*W. chitiniclastica* (96.9%)	*W. chitiniclastica* (99.7%)	*W. chitiniclastica* (75.0%)
105984	*W. chitiniclastica* (2.23)	*A. lwoffii* (96%)	*W. chitiniclastica* (99.6%)	*W. chitiniclastica* (99.7%)	*W. chitiniclastica* (75.0%)
106597	*W. chitiniclastica* (2.24)	*A. lwoffii* (96%)	*W. chitiniclastica* (99.9%)	*W. chitiniclastica* (99.8%)	*W. chitiniclastica* (75.0%)
108048	*W. chitiniclastica* (2.90)	*A. lwoffii* (96%)	*W. chitiniclastica* (99.9%)	*W. chitiniclastica* (99.3%)	*W. chitiniclastica* (75.1%)
108045	*W. chitiniclastica* (2.08)	*A. lwoffii* (96%)	*W. chitiniclastica* (99.6%)	*W. chitiniclastica* (100%)	*W. chitiniclastica* (74.9%)
110179	*W. chitiniclastica* (2.32)	*A. lwoffii* (96%)	*W. chitiniclastica* (99.4%)	*W. chitiniclastica* (99.4%)	*W. chitiniclastica* (75.0%)
110473	*W. chitiniclastica* (2.32)	*A. lwoffii* (92%)	*W. chitiniclastica* (99.7%)	*W. chitiniclastica* (99.1%)	*W. chitiniclastica* (74.0%)
18708^T^*	*W. chitiniclastica* (2.26)	*A. lwoffii* (96%)	*W. chitiniclastica* (99.9%)	*W. chitiniclastica* (99.4%)	*W. chitiniclastica* (100%)

*The type strain DSM 18708^*T*^ was included as reference.*

^T^*Type strain as reference.

### Identification of *W. chitiniclastica* Using MALDI-TOF MS

Identification of the strain collection of *W. chitiniclastica* using MALDI TOF MS was performed as previously described (Schröttner et al., 2014, 2016). In brief, strains were grown on Colombia blood agar plates for 24 h at 37°C. Single colonies were picked and plated on a 96-well steel target. Bacteria were dried on a laboratory workbench for 10 min and then overlaid with a 1 μl matrix (α-Cyano-4-hydroxycinnamic acid, Bruker Daltonik, Bremen, Germany) dissolved in an organic solvent. Subsequently, MALDI-TOF MS analyses were performed using flexControl software 3.1 (Bruker Daltonik, Bremen, Germany) following the manufacturer’s guidelines. Results are displayed in Table 3.

### Identification of *W. chitiniclastica* Using 16S rRNA Gene Analysis

Prior to PCR amplification, the performance of different primer pairs was evaluated *in silico* using TestPrime (Klindworth et al., 2012)^1^ based on the SILVA Reference database (release SSURef 138 NR) (Quast et al., 2013). PCR was carried out using the following primer pair combinations: (i) TPU-1 (5′-AGA GTT TGA TCM TGG CTC AG-3′) and RTU-4 (5′-TAC CAG GGT ATC TAA TCC TGT T-3′) (Funke et al., 2004); and (ii) 27F (5′-AGA GTT TGA TCM TGG CTC AG-3′) (Hongoh et al., 2003) and 1492R (5′-TAC CAG GGT ATC TAA TCC TGT T-3′) (Weisburg et al., 1991). 16S rRNA gene amplification was performed as previously described (Schröttner et al., 2014, 2016). Oligonucleotides were purchased from Biomers.net (Ulm, Germany). PCR products were purified using exonuclease I and shrimp alkaline phosphatase (both enzymes were purchased from New England Biolabs, Frankfurt am Main, Germany). Sanger sequencing was performed by SEQLAB (Sequence Laboratories Göttingen, Göttingen, Germany). Taxonomic identification was done based on BLASTN (Altschul et al., 1990), using the standard database for Nucleotide collection (nt). Results are displayed in Table 3.

### Whole Genome Analysis of *W. chitiniclastica*

**FIGURE 2:**
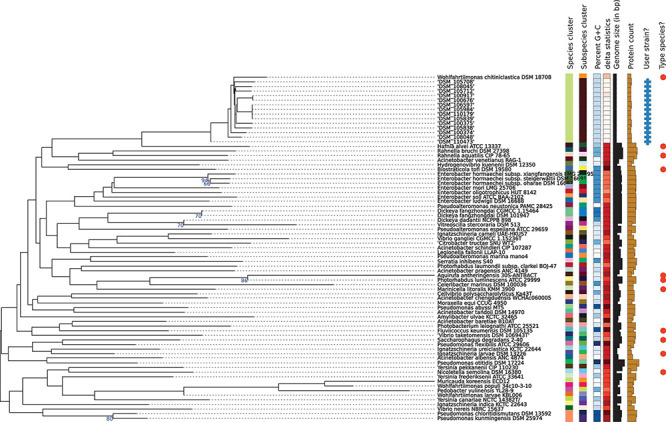
Phylogenomic tree of the *W. chitiniclastica* species and subspecies delineation based on the GBDP phylogenetic analyses retrieved from the TYGS website. The tree was inferred with FastME 2.1.6.1 (Lefort et al., 2015) from GBDP distances calculated from genome sequences and was subjected to a clustering using established thresholds for delineating species (DDH > 70%) (Meier-Kolthoff et al., 2013a) as well as subspecies (DDH > 79%) (Meier-Kolthoff et al., 2014). The branch lengths are scaled in terms of GBDP distance formula d5. The numbers above branches are GBDP pseudo-bootstrap support values >60% from 100 replications, with an average branch support of 83.0%.

Libraries for Whole Genome Sequencing (WGS) on the Illumina platform were prepared from extracted genomic DNA, applying the Nextera XT DNA Library Preparation Kit (Illumina, San Diego, United States) with modifications (Baym et al., 2015). Samples were sequenced on the NextSeq^TM^ 550 with a read length 2 × 150 bp targeting approx. 100× genome coverage followed by short read genome assembly using SpaD ES 3.14 (Bankevich et al., 2012). Whole genome sequences were submitted to NCBI GenBank under Acc. Nos JAGIBR000000000-JAGICE000000000, applying the NCBI Prokaryotic Annotation Pipeline PGAP (Tatusova et al., 2016). Contigs smaller than 300 bp were excluded from the submission. For phylogenomic identification and phylogenomic tree construction, genomic contigs were submitted to the Type Strain Genome Server (TYGS) at tygs.dsmz.de, and the type-based species clustering was done using a 70% dDDH threshold (Meier-Kolthoff and Göker, 2019). Subspecies clustering was based on a 79% dDDH threshold as previously introduced (Meier-Kolthoff et al., 2014). Results are displayed in Table 3 and Figure 2.

### Antibiotic Profiling

The antibiotic susceptibility testing of all *W. chitiniclastica* isolates was performed using MIC Test Strips (bestbion, Cologne, Germany) according to the manufacturer’s instructions. In brief, a McFarland standard of 0.5 was created for each strain, using NaCl and a DensiCHEK densitometer (bioMérieux, Nürtingen, Germany). The suspended bacteria were plated with a cotton swab on Müller-Hinton Agar (Oxoid Deutschland, Wesel, Germany). Then, MIC Test Strips (bestbion, Cologne, Germany) for each antibiotic were placed on the agar plates. The plates were incubated for 18 ± 2 h at 37°C. The MIC results were evaluated by applying the guidelines for PK/PD (non-species-related) breakpoints according to the criteria published by EUCAST (European Committee on Antimicrobial Susceptibility Testing), using Version 11.0, 01. January 2021.^2^ Results are displayed in Table 4. For the type strain DSM 18708^T^, results are represented in Supplementary Table 6. All MIC Test Strips used in this study and their MIC ranges are listed in Supplementary Table 2. The comprehensive antibiotic resistance database CARD^3^ was used for *in silico* prediction of antibiotic resistance genes (Alcock et al., 2020). Results are listed in Supplementary Tables 3, 4.

**TABLE 4:** Minimum inhibitory concentration (MIC) distribution of 14 *W. chitiniclastica* strains.

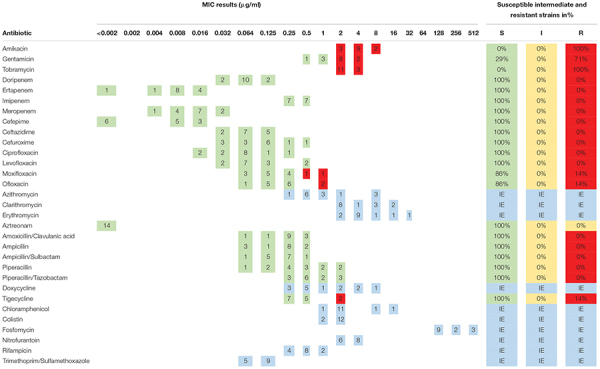

*This table summarizes the resistance profiles determined for 14 *W. chitiniclastica* strains. The MIC results are given in μg/ml. The number of isolates tested for each antibiotic is summarized in this table. Susceptible isolates are highlighted in green color, intermediate in yellow and resistant isolates in red. Blue color is used to illustrate the cases with insufficIEnt evidence (IE) that the antibiotic can successfully be administered to the patIEnt. In these instances, breakpoints are not provided by the EUCAST. Additionally, the percentages of susceptible and resistant strains are given.*

## Results

### Strain Collection and Its Characteristics

An overview of the *W. chitiniclastica* strain collection analyzed in this study is given in Table 2. All isolates were collected from wound swabs and patients’ medium age was 67.86 years ranging from 43 to 90 years. The majority were male (*n* = 13) and suffered from diabetes. There was only one female patient. This patient suffered from renal failure. Information regarding the social situation and living conditions were provided only in three cases (Schröttner et al., 2017). One patient (case 2) was homeless while one (case 3) lived under poor hygienic conditions. In contrast to these two, one patient (case 1) lived under normal conditions, which were not further specified. The associated microbial spectrum consists of *Staphylococcus* spp., *Pseudomonas* spp., *Proteus* spp., and *Streptococcus* spp. among others. Therefore, it remains unclear if *W. chitiniclastica* was the causative agent of the diseases or part of the microbiome.

### Identification of *W. chitiniclastica* Based on 16S rRNA Gene Analysis

16S rRNA amplification from pure culture of each isolate was successful and both primer pairs have proven of value (Table 2). In all cases sequence identity for *W. chitiniclastica* was above ≥98.7% and therefore fulfilling the criteria for bacterial species identification (Meier-Kolthoff et al., 2013b).

### Identification of *W. chitiniclastica* Based on MALDI-TOF MS

*Wohlfahrtiimonas chitiniclastica* was successfully identified by MALDI-TOF MS (Schröttner et al., 2016). Based on the manufacturer’s guidelines, score values above 2.0 are interpreted as secure identification at both the genus and species levels; scores between 2.0 and 1.7 as reliable identification on the genus, but not at the species level; and scores below 1.7 were regarded as an unreliable identification (Schröttner et al., 2016). In this study, all scores were above 2 (Table 2), allowing us to identify all isolates as *W. chitiniclastica* with high confidence.

### Results Obtained for Identification of *W. chitiniclastica* From VITEK 2

VITEK 2 results of *W. chitiniclastica* lead to misidentification as *Acinetobacter lwoffii* of all strains included in this study (Table 3), which has also been reported in previous case reports (de Dios et al., 2015; Chavez et al., 2017; Snyder et al., 2020). Identification of DSM 110473 resulted in 92% probability for species identification. Based on the manufacturer’s instructions 89–92% probability correlates with a good confidence level. Notably, for the remaining isolates, the results were above 96%, representing an excellent species identification; however, in our study a misidentification as *A. lwoffii* was evident for all strains. Biochemical characteristics based on the VITEK 2 system showed positive results for tyrosine arylamidase, Ellman’s reagent, L-lactate alkalization, and oxidase (Supplementary Table 5).

### Identification of *W. chitiniclastica* Based on Whole Genome Sequencing

Phylogenomic analysis of all 14 strains revealed correct taxonomic assignment to *W. chitiniclastica.* Hereby, dDDH values of 74.0–75.1% (Table 3) were computed against the type strain of the species DSM 18708^T^ and therefore fulfilling the criteria for bacterial species identification (Meier-Kolthoff and Göker, 2019). A phylogenomic tree based on whole-genome sequences was constructed using the TYGS web server^4^ (Figure 2) and all 14 isolates cluster in one subclade with the type strain DSM 18708^T^. Notably, the isolates from Dresden form a new subspecies using a 79% dDDH threshold (Meier-Kolthoff et al., 2014).

### Antibiotic Profiling

The susceptibility profile and the MIC distribution of all strains tested using MIC Test Strips are summarized in Table 4. Additionally, the MIC results of each isolate are provided in Supplementary Table 1. All 14 strains were susceptible to all penicillins, carbapenems, cephalosporines, and monobactams tested in this study. This is in line with the susceptibility profile of the type strain DSM 18708^T^ (Supplementary Table 5).

In addition, all 14 strains were susceptible to the fluoroquinolones tested in this study, apart from two exceptions. The isolates DSM 105984 and DSM 106597 were resistant to ofloxacin and moxifloxacin (Supplementary Table 1). Interestingly, these two strains showed higher MIC values for all tested fluoroquinolones in comparison to the other strains (Supplementary Table 1). A similar picture was obtained for the tetracycline tigecycline. The majority were susceptible while DSM 105984 and DSM 106597 appear to be resistant. In contrast, all strains appear to be resistant to the aminoglycosides amikacin and tobramycin. In addition, 10 strains showed resistance to gentamicin. The type strain DSM 18708^T^ appears to be susceptible to all three aminoglycosides tested in this study (Supplementary Table 5).

No breakpoints were available for trimethoprim/sulfamethoxazole, fosfomycin, doxycycline, colistin, chloramphenicol, nitrofurantoin, rifampicin, and macrolides. However, the MIC results determined for fosfomycin are all at a high range. Therefore, antimicrobial resistance to this antibiotic may be assumed. On the contrary, low MIC results were obtained for trimethoprim/sulfamethoxazole making susceptibility feasible.

The Comprehensive Antibiotic Resistance Database (CARD) (Alcock et al., 2020) was used for *in silico* identification of potential genes encoding for antibiotic resistance. Using the parameters “Perfect hit and Strict hit only” and “High-quality/coverage,” the strains DSM 100676, DSM 100917, and DSM 105708 each revealed one strict hit for *tet(D)* with an identity of 52.28% for the matching region (Supplementary Table 3). The “Strict” algorithm of the CARD system represents a flexible sequence variation but lies within the curated BLAST bit score cut-offs (Alcock et al., 2020) and by that making a correct identification still highly feasible. For the remaining isolates, no perfect or strict hits were obtained. However, when the parameter was changed to “Perfect hit, Strict hit, and Loose hit,” about 150 hits per strain were revealed with an identity for the matching region from 21 to 81%. Detailed results are displayed in Supplementary Table 3. However, keeping in mind that the “Loose” algorithm of the CARD system works outside of the detection model cut-offs to provide detection of new and more distant homologs of antimicrobial resistance (AMR) genes (Alcock et al., 2020), *in silico* results based on loose hits should always be taken with caution and require further research. Nevertheless, it could help us to identify potential resistance genes and/or shed light on new unknown modifications.

With respect to the resistance mechanism, the majority of hits belonged to antibiotic target alteration and efflux systems (Supplementary Table 4). Attention should be paid to genes mentioned as follows: Up to six genes encoding for fosfomycin efflux proteins were identified, and each strain showed a hit for MurA transferase with mutation conferring resistance to fosfomycin, which is involved in antibiotic target alteration of fosfomycin (Fu et al., 2015). In addition, several genes involved in broad range efflux systems of diverse antibiotics such as aminoglycosides, makrolides, fluoroquinolones, and tetracycline were detected including genes coding for macrolide-specific efflux pumps (*macA* and *macB*) (Kobayashi et al., 2000; Yum et al., 2009). Each strain also contained genes encoding for aminoglycoside resistant mechanisms such as *apmA, baeS*, and *cpxA*. Interestingly, all isolates revealed hits for the trimethoprim resistance gene *dfrI* (56.63% identity) and the sulfonamide resistance gene *sul3* (39.85% identity), as well as several hits for potential genes coding for β-lactamases, such as PNGM-1, NmcR, and GOB-16, although identity of the matching region was less than 36% in all cases.

## Discussion

The majority of the *W. chitiniclastica* isolates collected for the study was associated with chronic open skin wounds and related to comorbidities, such as diabetes. Moreover, poor social and hygienic conditions can be considered a risk factor for an infection with this human pathogen (Schröttner et al., 2017). These findings are in line with previous case studies, which further emphasize the correlation between the emergence of infectious diseases and social and economic inequalities (Campisi et al., 2015; de Dios et al., 2015; Suryalatha et al., 2015; Nogi et al., 2016; Chavez et al., 2017). Other microbial species were detected along with *W. chitiniclastica*, making it difficult to modulate the pathogenic potential of one or the other and/or appoint the causative agent of the infection in our collection. Although only rudimentary information is available and no thorough characterization of the microbial community took place, it is interesting to note that similarities to the diabetic foot microbiome occur. The genera *Staphylococcus*, *Pseudomonas* as well as *Streptococcus* were recently described as dominant taxa in chronic diabetic foot ulcers (Gardner et al., 2013; Wolcott et al., 2016; Gardiner et al., 2017) while *Proteus* spp. was specific to individuals (Gardiner et al., 2017). Although *W. chitiniclastica* occurred as part of polymicrobial infections (Campisi et al., 2015; de Dios et al., 2015; Kõljalg et al., 2015; Nogi et al., 2016), it has not yet been reported to be an abundant member of the diabetic skin and/or wound microbiome. However, further research could uncover its specific role and potential pathogenicity in association with chronic wounds and diseases, such as diabetes.

16S rRNA sequencing has proven to be a good and rapid identification method for bacterial organisms directly from clinical samples. However, the diagnostic power of this technique heavily depends on the choice of primer (Armougom, 2009; Klindworth et al., 2012) as well as the amplicon length and coverage of variable regions (Sune et al., 2020). Suboptimal primers can fail to detect single species or even whole groups (Andersson et al., 2008; Tringe and Hugenholtz, 2008; Wang and Qian, 2009), and the different variable regions within the 16S rRNA gene exhibit varying degrees of sequence diversity while no single region is able to distinguish among all bacteria (Chakravorty et al., 2007). Therefore, careful choice of primer pairing prior to amplification is strongly recommended. In this study, we decided to choose primer pairs, which are well-established in clinical routine diagnostics and satisfy with a good *in silico* coverage for the genus *Wohlfahrtiimonas*. Assuming that a standard PCR can tolerate up to two mismatches between the primer and its target (Nossa et al., 2010), the two primer pairs were chosen based on their TestPrime results (Klindworth et al., 2012) with an *in silico* coverage of 100%, and we are pleased to report that both primer pairs successfully amplified the 16S gene sequence of *W. chitiniclastica* from pure culture.

TPU-1/RTU-4 has been widely used in medical research as well as routine diagnostics (Funke et al., 2004; Haanperä et al., 2007; Broecker et al., 2016; Rudolph et al., 2019) and generates a shorter ∼800 bp fragment spanning hypervariable region V1 through V5, respectively. Hereby, V1-V3 is expected to provide reasonable taxonomic resolution to discriminate between taxa (Johnson et al., 2019) and enabled us to identify each isolate as *W. chitiniclastica*. However, it has been shown that taxonomic analysis based on short amplicons cannot achieve the taxonomic resolution afforded by sequencing the entire (∼1,500 bp) gene (Johnson et al., 2019), which still poses a problem for in-depth phylogenetic analysis (Tringe and Hugenholtz, 2008; Nossa et al., 2010; Johnson et al., 2019). The combination 27F/1492R overcomes this limitation by generating amplicons, spanning all nine hypervariable regions, which we would recommend when accurate classification of individual organisms at very high taxonomic resolution is required. In summary, TPU-1/RTU-4 as well as 27F/1492R is suitable for amplification of the 16S rRNA gene of *W. chitiniclastica* from pure culture and leads to correct identification. Although this has not been tested yet, we believe that both primer pairs are also suitable to detect *W. chitiniclastica* within the scope of a thorough microbial community profiling.

MALDI-TOF MS-based microbial identification is a well-established method in routine diagnostics (Mellmann et al., 2008; Schröttner et al., 2016). Although limitation on species level identification due to missing spectra in the database of unknown species might occur (Timperio et al., 2017; Strejcek et al., 2018), it has the advantage of speed and low cost, which most likely have priority in daily clinical practice (Seng et al., 2009). In case of an infection with *W. chitiniclastica* it excels as a fast and inexpensive identification tool.

The VITEK 2 system proved to be ineffective for identification of *W. chitiniclastica* isolates as previously reported (de Dios et al., 2015; Chavez et al., 2017; Snyder et al., 2020). The information about this bacterium appears to be missing the VITEK 2 database, leading to misidentification as *A. lwoffii.* Both organisms are Gram-negative, rod-shaped and show almost identical biochemical characteristics. Based on the VITEK 2 system, differences occur only with respect to the oxidase reaction. *A. lwoffii* is oxidase negative unlike *W. chitiniclastica* (Tóth et al., 2008; de Dios et al., 2015). Little is known about a potential natural habitat of *W. chitiniclastica* in humans apart from its association to parasitic flies, unlike *A. lwoffii*, which is described to be part of the physiological skin flora (Seifert et al., 1997; Berlau et al., 1999) but could also cause severe infections in humans (Ku et al., 2000). The latter could be problematic since *Acinetobacter* species are known for inherent resistance against many of the available antimicrobial agents (Tripathi et al., 2014), leading for example to multidrug-resistant clinical isolates of *A. lwoffii* (Hu et al., 2011). Although most *Acinetobacter* strains are still susceptible to carbapenems (Peleg et al., 2008), first resistances have emerged (Eliopoulos et al., 2008), making it a significant challenge to treat possible infections. *W. chitiniclastica*, on the other hand, appears to be susceptible to the majority of known antibiotics (Schröttner et al., 2017; Matos et al., 2019). Due to this contrary picture in terms of susceptibility for these two organisms, appropriate identification of the causing microorganisms and their resistance profiles is crucial to limit formation of multidrug-resistant species. With the currently available database, the VITEK 2 system appears not to be reliable and we therefore do not advocate for this method. For the identification of *W. chitiniclastica* isolates, we would rather recommend using MALDI-TOF MS and 16S rRNA gene sequencing. Although both serve as reliable identification tools, we would endorse a combination of both methods as it leads to a higher reliability and more robust identification accuracy (Schröttner et al., 2016), which is especially beneficial in case of doubtful results (Schröttner et al., 2014). Keeping in mind that neither MALDI-TOF MS nor 16S rRNA gene sequencing might be in use for routine diagnostics in every diagnostic laboratory, *W. chitiniclastica* might be even more common but misidentified due to a suboptimal diagnostic method. Moreover, with 23 human case reports from 18 different locations a clear geographical clustering appears to be missing (Table 1 and Figure 1) suggesting a potential spread and transmission. This further emphasize the hypothesis that *W. chitiniclastica* might be not as rare as originally anticipated.

Our study indicates that dDDH has proven a worthy identification method for *W. chitiniclastica*; however, since dDDH is a very costly and time-consuming technique and requires access to next generation sequencing technology (NGS), it is most likely irrelevant in daily clinical routine diagnostics. Notably, our 14 isolates from Dresden cluster in a subspecies, a fact that has not been described for any *W. chitiniclastica* strain yet. Subspecies are known to show adaptation to different environments (Biller et al., 2015; Lamas et al., 2018) and geographical locations (Truong et al., 2015; Costea et al., 2017). For example, recent studies of the human gut showed that discrete subspecies of the species *Agathobacter rectalis* and *Prevotella copri* are associated with geographically distinct human populations (Truong et al., 2015; Costea et al., 2017), whereas few strains occurred in multiple unrelated cohorts (Truong et al., 2015). Keeping in mind that the type strain DSM 18708^T^ has been isolated from animal source in Hungary (Tóth et al., 2008), the formation of a novel subspecies within the 14 isolates from Dresden most likely represents the adaptation to a human environment or a different geographic location. With hopefully increasing numbers of available genomes associated to human cases preferentially from various locations, large-scale genome analysis is recommended to unravel phenotypic and/or genotypic differences within the *W. chitiniclastica* clade.

*Wohlfahrtiimonas chitiniclastica* are described to be susceptible to the majority of known antibiotics with the exception of fosfomycin (Schröttner et al., 2017; Matos et al., 2019); however, no comprehensive and comparative antimicrobial resistance profiling of a larger strain collection has been performed so far. Based on our *in vitro* susceptibility testing, all *W. chitiniclastica* isolates appear to be susceptible to β-lactam antibiotics such as penicillins, cephalosporines, monobactams, and carbapenems. This is in contrast to the complete genome sequence analysis of the *W. chitiniclastica* strain BM-Y, which carried a *bla*_*VEB*__–__1_ gene cassette, thus conferring resistance to ceftazidime and ampicillin, among others (Zhou et al., 2016). Interesting to note is that our *in silico* analysis also revealed potential genes coding for β-lactamases such as PNGM-1 (Park et al., 2018), NmcR (Naas and Nordmann, 1994) and GOB-16 (Morán-Barrio et al., 2007); however, the identity of the matching region was less than 36% in all cases. Therefore, we believe that *W. chitiniclastica* contains at most an incomplete beta-lactamase or a homologous protein with a yet unknown function. This is in line with previous case studies, where cephalosporins, such as cefuroxime, have proven to be successful to treat infections caused by *W. chitiniclastica* (Rebaudet et al., 2009; Campisi et al., 2015; Suryalatha et al., 2015; Snyder et al., 2020; Bueide et al., 2021), and by that supporting our results of a comprehensive susceptibility against a wide majority of β-lactam antibiotics.

All 14 strains were susceptible to the fluoroquinolones ciprofloxacin and levofloxacin. This is congruent with two case studies, where an infection caused by *W. chitiniclastica* was successfully treated with levofloxacin (Schröttner et al., 2017; Bueide et al., 2021) as well as brief antibiotic susceptibility tests within individual clinical reports (de Dios et al., 2015; Kõljalg et al., 2015; Chavez et al., 2017; Katanami et al., 2018; Snyder et al., 2020). In this study, the majority of the isolates was also susceptible to moxifloxacin and ofloxacin apart from two exceptions. The isolates, DSM 105984 and DSM 106597, appear to be resistant. Interestingly, based on our *in silico* analysis, all isolates contained a point mutation in *gyrB* (58.73% identity), known to confer resistance to moxifloxacin in *Clostridioides difficile* (Walkty et al., 2010). However, a previous study showed that not all mutations leading to amino acid substitution in GyrB seem to be relevant, as at least one was also detected in susceptible strains (Spigaglia et al., 2008). Further research will be necessary to gain a better understanding whether this point mutation plays any role in resistance or not. Our *in silico* results could serve as basis for identifying potential target genes for a thorough sequence analysis and/or genetic mutations. Until then, ciprofloxacin and levofloxacin may be the best fluoroquinolones to use.

*In vitro* analysis with respect to tetracycline resistances showed a rather diverse picture. We obtained diversified MIC results for doxycycline, ranging from 0.35 up to 8 μg/ml. Unfortunately, no EUCAST guideline is available, and to the best of our knowledge, no case reports have been published so far. In the case of tigecycline, 12 isolates were susceptible while DSM 105984 and DSM 106597 were resistant. This rather diverse resistance profile for tetracycline is also reflected in the literature. In some case studies, the isolate was susceptible to tetracyclines (Almuzara et al., 2011; Nogi et al., 2016), and in another report, it was resistant (Snyder et al., 2020). This rather varying picture continues within the present *in silico* study. Resistance to tetracyclines can be governed by *tet* genes, such as *tet(D)*, which encode for a tetracycline antibiotic efflux pump (Levy et al., 1999; Hedayatianfard et al., 2014). Interestingly, the strains DSM 100676, DSM 100917, and DSM 105708 revealed one strict hit each for *tet(D)* with an identity of 52.28% for the matching region. Unfortunately, this is not congruent with the *in vitro* results for tigecycline, in which the strains DSM 100676, DSM 100917, and DSM 105708 were susceptible. Interestingly, for doxycycline, these three strains showed comparatively high MIC values with 4 and 8 μg/ml, respectively (Supplementary Table 1). Keeping in mind that the “Strict” algorithm of the CARD system represents a flexible sequence variation but lies within the curated BLAST bit score cut-offs (Alcock et al., 2020), resistance to doxycycline governed by *tet(D)* among others therefore appears to be feasible. The two tigecycline resistant isolates, DSM 106584 and DSM 106597, on the other hand, had no hits to *tet(D)* but loose hits to several efflux pumps and to a gene encoding for the mobile Tet(X) ortholog, which is described to confer high-level tigecycline resistance (Fang et al., 2020). However, since we only found loose hits, these *in silico* results should be taken with caution and require further research. This leaves us with a rather questionable picture regarding the tetracycline resistance profile, which still needs to be resolved.

Aminoglycosides (AG), such as amikacin, gentamicin, and tobramycin, are broad-spectrum antibiotics and interfere with the bacterial protein translation by binding to the bacterial ribosome. Common AG resistance mechanism include modification of the AG binding site by 16S rRNA methyltransferases (RMTases) and antibiotic target alteration by aminoglycoside phosphotransferases (APHs), aminoglycoside nucleotidyltransferases (ANTs), and aminoglycoside acetyltransferases (AACs) (Yokoyama et al., 2003; Fessler et al., 2011). Based on our *in silico* analysis, all strains contain a homolog to the *apmA* gene encoding for an AAC with 47.92% identity for the matching region. In addition, several hits for efflux pumps specific for aminoglycoside and aminocoumarin, such as *baeS* (Baranova and Nikaido, 2002; Nishino et al., 2005) and *cpxA* (Srinivasan et al., 2012), were identified, but the result should be viewed with caution since only loose hits are present and the percentage of the matching region was comparatively low, with 27.93 and 33.45%, respectively. Nevertheless, the *in vitro* experiments support the *in silico* analysis, which showed that all strains were resistant to amikacin and tobramycin. Similar results were obtained for the type strain DSM 18708^T^ (Supplementary Table 5), suggesting a natural aminoglycoside resistance profile. Moreover, other case studies detected analogous MIC results for amikacin and tobramycin (Kõljalg et al., 2015; Chavez et al., 2017; Katanami et al., 2018), but the interpretation of antibiotic susceptibility was based on the MIC breakpoints for other non-*Enterobacteriaceae* described in the M100 Performance Standards for Antimicrobial Susceptibility Testing, determined by the Clinical and Laboratory Standards Institute (CLSI) (Wayne and Clinical and Laboratory Standards Institute (CLSI), 2016). Therefore, those isolates were described as susceptible. Although previous studies showed an acceptable level of comparability between EUCAST and CLSI (Kassim et al., 2016; Akdoğan et al., 2021), one should always keep in mind that even small differences might influence the therapeutic option. We rather consider *W. chitiniclastica* as resistant to amikacin and tobramycin and would therefore not recommend aminoglycosides as first line treatment. However, reevaluation is strongly recommended when updated EUCAST breakpoints become available.

No EUCAST guidelines exist for trimethoprim/sulfamethoxazole, rifampicin, nitrofurantoin, fosfomycin, colistin, doxycyclin, erythromycin, azithromycin, and clarithromycin. However, for some antimicrobial substances, the *in vitro* analysis revealed comparatively low or high MIC values, respectively. This allows us to generate a hypothesis regarding the resistance profile for some isolates. For instance, we observed comparatively low MIC values for trimethoprim/sulfamethoxazole. This is in line with recent case reports, in which *W. chitiniclastica* were susceptible to trimethoprim/sulfamethoxazole (Chavez et al., 2017; Katanami et al., 2018; Connelly et al., 2019; Snyder et al., 2020; Bueide et al., 2021). Interestingly, all isolates revealed *in silico* hits for the trimethoprim resistant gene *dfrI* (Welch et al., 2007) (56.63% identity) and the sulfonamide resistance gene *sul3* (Perreten and Boerlin, 2003) (39.85% identity), assuming that resistance may be feasible. However, previous studies of *Escherichia coli* reported that trimethoprim resistant genes were not expressed due to defective promotors (Mazurek et al., 2015). Therefore, we believe that susceptibility toward trimethoprim/sulfamethoxazole of all isolates tested in this study appears to be feasible, despite the presence of potential resistance genes. Nevertheless, antimicrobial resistome analysis prior to treatment is always recommended as newly emerged resistance might arise quickly. For example, the first reported case of *W. chitiniclastica* infection in South Africa surprised with trimethoprim/sulfamethoxazole resistance (Hoffmann et al., 2016). To the best of our knowledge, this is the first report in which an isolate appears to be resistant.

Moderate MIC values for clarithromycin and erythromycin in combination with *in silico* detection of homologs genes coding for macrolide-specific efflux pumps (*macA* and *macB*) (Kobayashi et al., 2000; Yum et al., 2009) make resistance feasible. This is congruent with a preliminary genome report of a *W. chitiniclastica* strain, which also contained *macA* and *macB* genes (Matos et al., 2019). Nevertheless, further research is still required to uncover the macrolide resistance profile fully.

Finally, yet importantly, our *in vitro* analysis showed very high MIC values for fosfomycin for all isolates. This is in line with previous reports (Schröttner et al., 2017; Matos et al., 2019), making natural resistance very likely. Based on our *in silico* analysis, all isolates contained a gene homolog encoding for a potential MurA transferase with mutation conferring resistance to fosfomycin (Fu et al., 2015) (50.48% identity) and for the fosfomycin modifying glutathione transferase FosC2 (Wachino et al., 2010) (26.62% identity), among several other hits for efflux proteins. In addition, DSM 110473 carried with 62.67% identity a gene homologous to *mdtG*, which has been reported, when overexpressed, to increase fosfomycin resistances (Nishino and Yamaguchi, 2001). We strongly believe that *W. chitiniclastica* features a natural fosfomycin resistance, and maybe a yet unknown resistance mechanism might be present since no strong hits with high percentage identity were detected.

In conclusion, *W. chitiniclastica* has recently been described as a rare but potential new emerging human pathogen. However, with intensive usage of MALDI-TOF MS and 16S rRNA gene for identification, it might turn out that this species is even more common than currently anticipated. In case of infection, trimethoprim/sulfamethoxazole, levofloxacin, and cephalosporins, such as cefuroxime, may be the best antibiotics to use. Keeping in mind that exposure of many antibiotics lead to enormous selective pressures including resistome expansion (Wright, 2007), constant reevaluation is strongly recommended, in particular when updated EUCAST guidelines become available and/or new case studies are published. Further research is also required in order to identify genes or mutations that are responsible for antimicrobial resistance. The results of our *in silico* analysis could offer advantages in order to identify potential candidates for target specific manipulations, which will be a crucial component to unravel the genetic resistance profile of *W. chitiniclastica*.

## Data Availability Statement

The datasets presented in this study can be found in online repositories. The names of the repository/repositories and accession number(s) can be found below: https://www.ncbi.nlm.nih.gov/genbank/, JAGIBR000000000; JAGIBS000000000; JAGIBT000000000; JAGIBU000000000; JAGIBV000000000; JAGIBW000000000; JAGIBX000000000; JAGIBY000000000; JAGIBZ000000000; JAGICA000000000; JAGICB000000000; JAGICC000000000; JAGICD000000000; JAGICE000000000.

## Ethics Statement

The study was approved by the Ethics Committee at the Technical University of Dresden (EK 61022019). Written informed consent was not obtained from the individual(s) for the publication of any potentially identifiable images or data included in this article.

## Author Contributions

PS had the idea and the concept for the study. AK performed the experiments, analyzed the data, and wrote the first version of the manuscript. BB provided the bioinformatic data from the whole genome sequences. TR performed the curation of the bacteria and the inclusion into the DSMZ “Open Collection”. BB, TR, SC, FG, and PS contributed text passages for the manuscript. All authors contributed to the revision of the manuscript and approved the present version.

## Conflict of Interest

The authors declare that the research was conducted in the absence of any commercial or financial relationships that could be construed as a potential conflict of interest.

## Publisher’s Note

All claims expressed in this article are solely those of the authors and do not necessarily represent those of their affiliated organizations, or those of the publisher, the editors and the reviewers. Any product that may be evaluated in this article, or claim that may be made by its manufacturer, is not guaranteed or endorsed by the publisher.

## Footnotes

1
https://www.arb-silva.de/search/testprime/

2
http://www.eucast.org/clinical_breakpoints/

3
https://card.mcmaster.ca/

4
https://tygs.dsmz.de/
