# Supplementary material for: Identification and Antibiotic Profiling of Wohlfahrtiimonas chitiniclastica, an Underestimated Human Pathogen
Source: Front Microbiol. 2021 Sep 22;12:712775. doi: 10.3389/fmicb.2021.712775 (PMC8496446; doi:10.3389/fmicb.2021.712775)
Supplement: Supplementary file 1 [file Data_Sheet_1.PDF]

Table S1: MIC values in µg/ml for the 14 *W. chitiniclastica* isolates tested in this study.

	DSM number													
**Antibiotic**	**100374**	**100375**	**100676**	**100917**	**105708**	**105712**	**105838**	**105839**	**105984**	**106597**	**108045**	**108048**	**110179**	**110473**
**Ampicillin/Sulbactam**	0.25	0.125	0.25	0.064	0.125	0.25	0.25	0.125	0.5	0.25	0.125	0.25	0.125	0.25
**Cefuroxim**	0.125	0.125	0.125	0.125	0.064	0.064	0.032	0.125	0.5	0.25	0.032	0.064	0.032	0.125
**Azithromycin**	0.5	1	0.5	0.5	1	0.5	1	2	8	8	0.5	8	0.5	0.25
**Piperacillin/Tazobactam**	0.5	0.5	1	2	0.5	0.5	1	0.25	2	2	0.25	0.5	0.25	0.5
**Doripenem**	0.064	0.064	0.064	0.125	0.064	0.064	0.125	0.064	0.064	0.064	0.032	0.64	0.032	0.064
**Trimethroprim/Sulfamethoxazole**	0.064	0.125	0.125	0.064	0.125	0.125	0.125	0.064	0.125	0.125	0.064	0.125	0.064	0.125
**Ceftazidim**	0.125	0.064	0.125	0.064	0.064	0.064	0.032	0.064	0.125	0.125	0.032	0.064	0.064	0.125
**Tigecylin**	0.5	0.25	0.25	0.25	0.25	0.5	0.5	0.25	2	2	0.5	0.25	0.5	0.25
**Nitrofurantoin**	4	2	2	2	4	2	4	4	4	4	4	2	2	4
**Tobramycin**	2	2	4	4	2	2	4	2	2	2	2	2	2	2
**Ciprofloxacin**	0.064	0.064	0.064	0.064	0.032	0.064	0.064	0.064	0.125	0.25	0.016	0.032	0.016	0.064
**Rifampicin**	0.5	0.5	0.25	0.25	1	0.5	0.25	0.25	0.5	1	0.5	0.5	0.5	0.5
**Ofloxacin**	0.125	0.25	0.25	0.25	0.064	0.25	0.25	0.25	1	1	0.125	0.125	0.125	0.125
**Colistin**	2	2	2	1	2	2	2	2	2	2	2	2	1	2
**Amoxicillin-Clavulansäure**	0.25	0.25	0.5	0.25	0.25	0.25	0.25	0.25	0.5	0.5	0.064	0.25	0.125	0.25
**Clarithromycin**	8	2	2	2	8	2	8	4	16	16	2	2	2	2
**Aztreonam**	0	0	0	0	0	0	0	0	0	0	0	0	0	0
**Ampicillin**	0.25	0.125	0.25	0.25	0.25	0.25	0.25	0.25	0.5	0.5	0.064	0.064	0.064	0.25
**Levofloxacin**	0.064	0.125	0.125	0.064	0.032	0.125	0.064	0.064	0.5	0.5	0.032	0.064	0.064	0.064
**Moxifloxacin**	0.064	0.125	0.25	0.25	0.125	0.25	0.25	0.125	0.5	1	0.064	0.125	0.064	0.125
**Piperacillin**	0.5	0.25	1	1	0.25	0.25	0.064	0.5	2	2	0.125	0.25	0.125	0.5
**Imipenem**	0.5	0.25	0.25	0.5	0.5	0.5	0.5	0.5	0.25	0.25	0.25	0.5	0.25	0.25
**Fosfomycin**	512	512	128	128	512	128	256	128	128	128	128	128	128	256
**Gentamicin**	1	2	4	4	2	2	0.5	1	2	2	2	2	1	2
**Cefepim**	0.016	0	0.032	0.016	0	0	0.032	0	0.032	0.032	0.032	0.016	0	0.016
**Amikacin**	4	4	2	8	4	4	8	4	4	4	2	4	2	4
**Erythromycin**	4	4	4	2	8	4	4	4	32	12	4	2	4	4
**Ertapenem**	0.008	0.008	0.008	0.016	0.008	0.016	0.016	0.008	0.008	0.008	0.004	0.008	0	0.016
**Doxycyclin**	0.5	0.25	4	4	8	1	0.5	0.5	2	2	0.5	0.25	0.25	0.5
**Meropenem**	0.016	0.016	0.032	0.032	0.008	0.016	0.016	0.016	0.008	0.008	0.008	0.016	0.004	0.016
**Chloramphenicol**	2	2	2	1	2	2	2	2	8	16	2	2	2	2

Table S2: Antibiotics used and MIC ranges covered.

Antibiotic	MIC range µg/ml
Amikacin	0.016-256
Gentamicin	0.064-1024
Tobramycin	0.016-256
Doripenem	0.002-32
Ertapenem	0.002-32
Imipenem	0.002-32
Meropenem	0.002-32
Cefepim	0.016-256
Ceftazidim	0.016-256
Cefuroxim	0.016-256
Ciprofloxacin	0.002-32
Levofloxacin	0.002-32
Moxifloxacin	0.002-32
Ofloxacin	0.002-32
Azithromycin	0.016-256
Clarithromycin	0.016-256
Erythromycin	0.016-256
Aztreonam	0.016-256
Amoxicillin-Clavulansäure	0.016-256
Ampicillin	0.016-256
Ampicillin/Sulbactam	0.016-256
Piperacillin	0.016-256
Piperacillin/Tazobactam	0.016-256
Doxycyclin	0.016-256
Tigecylin	0.016-256
Chloramphenicol	0.016-256
Colistin	0.016-256
Fosfomycin	0.064-1024
Nitrofurantoin	0.032-512
Rifampicin	0.016-256
Trimethroprim/Sulfamethoxazole	0.002-32

Table S4: Number of hits per resistance mechanism based on the CARD analysis

	DSM number													
**Resistance mechanisms**	**100374**	**100375**	**100676**	**100917**	**105708**	**105712**	**105838**	**105839**	**105984**	**106597**	**108045**	**108048**	**110179**	**110473**
Antibiotic inactivation	4	4	4	4	4	4	4	4	4	4	4	4	4	4
Antibiotic target replacement	5	5	5	5	5	5	5	5	5	5	4	5	5	5
Antibiotic target protection	4	4	5	5	5	5	4	5	5	4	5	4	5	4
Antibiotic target alteration	31	32	33	32	32	32	31	31	31	31	32	31	31	32
Reduced permeability to antibiotic	6	6	6	6	6	6	6	7	7	7	6	6	7	6
Antibiotic efflux	83	80	82	82	79	84	80	79	79	79	84	81	79	83

Table S5: Obtained VITEK 2 results using the card for identification of Gram-negative organisms (GN). The substrates on this card are listed in Table 7 of the Encyclopedia of Rapid Microbiological Methods

**DSM number**	**VITEK 2 identification results**	**Biocgemical characteristics**
100374	*A. Iwoffii*	OX, TyrA, ILATa, ELLM
100375	*A. Iwoffii*	OX, TyrA, ILATa
100676	*A. Iwoffii*	OX, TyrA, ILATa, ELLM
100917	*A. Iwoffii*	OX, TyrA, ILATa, ELLM
105708	*A. Iwoffii*	OX, TyrA, ILATa, ELLM
105712	*A. Iwoffii*	OX, TyrA, ILATa, ELLM
105838	*A. Iwoffii*	OX, TyrA, ILATa, ELLM
105839	*A. Iwoffii*	OX, TyrA, ILATa, ELLM
105984	*A. Iwoffii*	OX, TyrA, ILATa, ELLM
106597	*A. Iwoffii*	OX, TyrA, ILATa, ELLM
108048	*A. Iwoffii*	OX, TyrA, ILATa, ELLM
108045	*A. Iwoffii*	OX, TyrA, ILATa, ELLM
110179	*A. Iwoffii*	OX, TyrA, ILATa, ELLM
110473	*A. Iwoffii*	OX, TyrA, ILATa, ELLM

OX = Oxidase, TyrA = Tyrosine Arylamidase, ILATa = L-Lactate Alkalinization, ELLM = Ellman

Table S6: MIC results (µg/ml) for the type strain 18708 of *Wohlfahrtiimonas chitiniclastica*. Susceptible is highlighted in green color, intermediate in yellow, and resistant isolates in red. Blue color is used to illustrate the cases with insufficient evidence (IE) that the antibiotic can successfully be administered to the patient, In these instances breakpoints are not provided by the EUCAST.

**Antibiotic**	**MIC (mg/ml)**
Amikacin	4
Gentamicin	2
Tobramycin	2
Doripenem	0.032
Ertapenem	0.008
Imipenem	0.25
Meropenem	0.016
Cefepim	0
Ceftazidim	0.064
Cefuroxim	0.125
Ciprofloxacin	0.032
Levofloxacin	0.064
Moxifloxacin	0.25
Ofloxacin	0.064
Azithromycin	0.5
Clarithromycin	2
Erythromycin	2
Aztreonam	0
Amoxicillin-Clavulansäure	0.125
Ampicillin	0.064
Ampicillin/Sulbactam	0.064
Piperacillin	0.25
Piperacillin/Tazobactam	0.5
Doxycyclin	0.5
Tigecylin	0.25
Chloramphenicol	2
Colistin	2
Fosfomycin	512
Nitrofurantoin	4
Rifampicin	0.5
Trimethroprim/Sulfamethoxazole	0.125
